# Correlative factors of ocular surface lesions after allogeneic hematopoietic stem cell transplantation: A retrospective study

**DOI:** 10.3389/fonc.2022.1040679

**Published:** 2022-11-21

**Authors:** Xin-Yu Zhuang, Zheng-Tai Sun, Yue Xu, Ya-Ru Ren, Ying-Jie Chen, Feng Chen, Xiao Ma, Xiao-Wen Tang, Xiao-Feng Zhang

**Affiliations:** ^1^ Department of Ophthalmology, First Affiliated Hospital of Soochow University, Suzhou, China; ^2^ Department of Ophthalmology, Dushu Lake Hospital Affiliated to Soochow University, Suzhou, China; ^3^ National Clinical Research Center for Hematologic Diseases, Jiangsu Institute of Hematology, The First Affiliated Hospital of Soochow University, Suzhou, China; ^4^ Institute of Blood and Marrow Transplantation, Collaborative Innovation Center of Hematology, Soochow University, Suzhou, China

**Keywords:** allogeneic hematopoietic stem cell transplantation, graft-versus-host disease (GVHD), ocular, ocular surface, keratoconjunctivitis sicca (KCS)

## Abstract

**Background:**

Ocular graft-versus-host disease (oGVHD) is one of the complications after allogeneic hematopoietic stem cell transplantation (HSCT), which impairs the quality of life and may indicate poor prognosis. In this retrospective study, the aim was to investigate the characteristics of ocular surface after HSCT, and analyze the risk factors related to the severity of ocular surface lesions.

**Methods:**

248 post-HSCT patients were enrolled in this retrospective study. Subjects were divided into no lesion group, mild lesion group and severe lesion group, according to the severity of ocular surface lesions. The correlations between grades of ocular surface lesions and gender, age, primary disease, donor source, human leukocyte antigen (HLA) type, kinship, donor-recipient relationship, blood type, source of stem cell and systemic GVHD were analyzed.

**Results:**

The median scores of corneal epitheliopathy, lid margin lesions and meibomian gland loss were 3, 6 and 2 points, respectively. The grade of corneal epitheliopathy was related to donor source (P<0.001), kinship (P=0.033), HLA-matching (P<0.001), and systemic GVHD (P=0.007), especially oral GVHD (P<0.001) and liver GVHD (P=0.002). The grade of lid margin lesions was related to donor source (P=0.019), HLA-matching (P=0.006), and systemic GVHD (P=0.013), especially skin GVHD (P=0.019) and oral GVHD (P=0.019). The grade of meibomian gland loss was related to age (P=0.035) and gastrointestinal GVHD (P=0.007). The grade of corneal epitheliopathy after HSCT was related to the lid margin lesion score (P<0.001).

**Conclusions:**

The occurrence and development of ocular GVHD are mostly accompanied by the history of systemic GVHD. While in few cases, ocular surface lesions related to GVHD can be observed prior to the rejection of other tissues and organs. Severe corneal epitheliopathy occurs in patients with severe lid margin lesions in ocular GVHD. The lesions of corneal epithelium and lid margin are milder in HLA partially matching transplantation.

## Introduction

Allogeneic hematopoietic stem cell transplantation (HSCT) is a common treatment for various malignant and non-malignant blood diseases. However, graft-versus-host disease (GVHD) is a common complication after HSCT (1, 2). The 2014 National Institutes of Health (NIH) consensus classified GVHD into acute GVHD and chronic GVHD according to the clinical features. Chronic GVHD is a syndrome of variable clinical features, which simultaneously involves more than one tissue and organ. These include skin, oral mucosa, liver, gastrointestinal tract, lung, joint and eyes. The diagnosis of chronic GVHD requires at least one diagnostic manifestation of chronic GVHD or at least one distinctive manifestation plus a pertinent biopsy, laboratory, or other tests (e.g., Schiemer test) in the same or another organ (3). The risk factors of GVHD mainly include age of donor and recipient, human leukocyte antigen (HLA) type, gender relation (especially transplantation between female donor and male recipient), source of stem cell (bone marrow or peripheral blood) and acute GVHD (4, 5).

Chronic GVHD has been largely divided into “limited” and “extensive”. Ocular GVHD (oGVHD) belongs to “extensive” chronic GVHD (2), and is seen in 60% to 90% of patients with GVHD (6). Ocular GVHD involves lacrimal gland, palpebra, and ocular surfaces, such as cornea, conjunctiva and meibomian glands. Keratoconjunctivitis sicca (KCS) is the most common symptom of oGVHD, and occurs in 40-60% of oGVHD cases (7). This impairs the quality of life and may indicate poor prognosis (8). Meibomian gland dysfunction (MGD) and blepharitis may also occur (9). Earlier it was assumed that oGVHD usually occurs along with systemic GVHD. However, it may have an isolated occurrence and can be long-lasting (10).

In recent times, the diagnosis of oGVHD mainly focuses on subjective symptoms, keratopathy and tear secretion, with limited focus on other ocular surface structures (3, 11). The aim of this study was to discover the characteristics of ocular surface, and analyze the factors related to the severity of ocular surface lesions after HSCT. In order to achieve this aim, we evaluated corneal epitheliopathy, lid margin lesions and meibomian gland loss after HSCT quantitatively.

## Materials and methods

### Subjects

We collected data of patients who underwent HSCT, and visited the department of ophthalmology in the First Affiliated Hospital of Soochow University and Dushu Lake Public Hospital Affiliated to Soochow University. This study was approved by the ethics committee of the First Affiliated Hospital of Soochow University, and adhered to the tenets of the Declaration of Helsinki (No.2022-235). Patient information was anonymized. The information of age, gender, primary disease, donor source, HLA type, kinship, gender consistence, blood type, source of stem cell and the history of systemic GVHD was recorded.

In this study, we included patients who met the following inclusion criteria: (1) diagnosed with hematologic disease by the hematology department, and underwent HSCT with stable vital signs at present; (2) with ocular symptoms, such as dry eyes, photophobia and foreign body sensation, after HSCT; (3) diagnosed with ocular GVHD. Criteria for chronic oGVHD: new ocular sicca documented by low Schirmer’s test with a mean value of ≤5mm at 5 minutes or a new onset of KCS detected by slit lamp exam with mean Schirmer test values of 6 to 10mm (3). The patients with following features were excluded: (1) uncontrolled systemic infections; (2) intraocular diseases like glaucoma, uveitis and intraocular infections; (3) with eyelid closure difficulty, trichiasis, entropion and ectropion; (4) with history of ocular herpes simplex virus and/or varicella-zoster virus infections; (5) with history of ocular operations.

### Ocular assessments

The right eye of each patient was examined and evaluated for tear break-up time (TBUT), Schirmer I test, corneal fluorescence staining (CFS), assessment of lid margin lesions, infrared imaging of meibomian gland. The images of ocular surface and lid margin were recorded and scored by the same doctor.

### Assessment and grading of corneal epitheliopathy

The ocular surface was stained with 1% sodium fluorescein dye, and then observed and photographed under the cobalt blue light of the slit lamp after one minute. No eye drops were used for two hours prior to assessment. The scoring method adopted was as suggested by Rose-Nussbaumer et al. (12). No corneal epithelium staining scored 0, 1-5 staining dots scored 1, 6-30 staining dots scored 2, and >30 staining dots scored 3. Moreover, an additional point was added for each of the following: corneal staining fusion, pupillary staining, and presence of one or more filaments. Subjects with score 0 were categorized in the no lesion group (C_0_), with 1-3 points in the mild corneal epitheliopathy group (C_1_), and those with 4-6 points in severe corneal epitheliopathy group (C_2_).

### Assessment and grading of lid margin lesions

The assessment of lid margin lesions was done under the diffused light of the slit lamp. The scores for hyperemia, irregularity, meibum quality, meibum secretion and gland obstruction were summed up to 14 points, and the total score was recorded. The scoring method by Arita et al. (13) was used for hyperemia and irregularity. In contrast, the method by Srinivasan et al. (14) was used for meibum quality, meibum secretion and gland obstruction (**Table 1**). Subjects with score 0 were categorized in the no lesion group (L_0_), with 1-7 points in the mild lid margin lesion group (L_1_), and those with 8-14 points in the severe lid margin lesion group (L_2_).

**Table 1:** Scales of lid margin lesions.

Score	Features of Lid Margin
Hyperemia	
0	no or mild hyperemia
1	eyelid hyperemia without telangiectasis
2	telangiectasia observed at the palpebral margin but do not cross the palpebral glandular foramen
3	telangiectasia seen at the palpebral margin and passing through the palpebral glandular foramen
Irregularity	
0	smooth eyelid margin without indentation
1	< 3 shallow indentations
2	≥ 3 shallow indentations or with deep indentation
Meibum quality	
0	clear meibum
1	turbid meibum
2	granular meibum
3	meibum concentrated like toothpaste or cannot be extruded
Meibum secretion	
0	clear meibum readily expressed
1	cloudy meibum expressed with mild pressure
2	cloudy meibum expressed with more than moderate pressure
3	meibum could not be expressed even with strong pressure
Gland obstruction	
0	no plugging
1	< 3 plugging
2	≥ 3 plugging with a distribution of less than half of the full length of the lid
3	≥ 3 plugging with a distribution of half or more of the full length of the lid

### Assessment and grading of meibomian gland loss

The infrared imaging instrument was used to obtain images of meibomian glands. The method by Arita et al. (15) was used to quantitate the loss of meibomian gland. Subjects with no loss of meibomian glands scored 0, those with area loss <1/3 of the total meibomian gland area scored 1, between 1/3 and 2/3 scored 2, and >2/3 scored 3. Scores for the upper and lower eyelids were summed to obtain the total score for each eye. Patients with score 0 were enrolled in no lesion group (G_0_), with 1-3 points in mild meibomian loss group (G_1_), and those with 4-6 points in severe meibomian loss group (G_2_).

### Analysis of factors related to ocular surface lesions

Correlation analysis was done between ocular surface lesions and patient information, such as gender, age, primary disease, donor source, HLA type, kinship, gender consistence, blood type, source of stem cell and history of systemic GVHD among the groups (C_0_, C_1_, C_2_; L_0_, L_1_, L_2_; G_0_, G_1_, G_2_; respectively).

### Statistical analysis

SPSS 26.0 statistical software (IBM Corp., New York, USA) was used in this study. Clinical data, such as gender, primary disease, donor source, HLA type, kinship, gender consistence, blood type and source of stem cell, were summarized as percentages. Ophthalmological data evaluation, including scores of CFS, lid margin lesions, and meibomian gland loss, were summarized as median (inter-quartile range) since Kolmogorov-Smirnov test proved that the distribution is not normal. Kruskal-Wallis test and pair-wise comparations were performed to compare measurement data among groups. Chi-squared test was used to compare enumeration data. Fisher exact test was applied if the theoretical number was less than 5. Further multiple regression analysis was performed for statistically significant factors. A P-value of<0.05 was considered to be statistically significant.

## Results

### Patient characteristics

As shown in **Table 2**, 248 subjects were enrolled in this study, of which 151 were males (61%) and 97 were females (39%). The mean age of patients was 34.66 ± 12.30 years. The average interval between HSCT and first visit to the ophthalmology department was 19.38 ± 18.20 months.

**Table 2:** Demographics and transplant characteristic.

	Results		Results
**Gender**		**Gender relationship**	
Male	151 (61)	Male-to-male	68 (29)
Female	97 (39)	Female-to-female	39 (17)
**Age(year)**		Male-to-female	55 (23)
	34.66 ± 12.30	Female-to-male	74 (31)
**Post-HSCT time(month)**		**Source of stem cell**	
	19.38 ± 18.20	BMT	17 (7)
**Primary disease**		PBSCT	107 (47)
ALL	71 (29)	BMT+PBSCT	106 (46)
AML	113 (46)	**Systemic GVHD**	
CML	16 (6)	With	196 (94)
MDS	26 (10)	Without	12 (6)
others	22 (9)	**Acute GVHD**	
**Donor source**		With	85 (49)
Haplo-HSCT	124 (50)	Without	87 (51)
Sib-HSCT	97 (39)	**Organs with GVHD**	
URD-HSCT	27 (11)	Skin	151 (73)
**Kinship**		Oral mucosa	95 (46)
Related	221 (89)	Liver	101 (49)
Unrelated	27 (11)	Gastrointestinal tract	49 (23)
**HLA**		Lung	28(13)
Fully match	123 (49.6)	Spleen	3 (1)
Partially match	125 (50.4)	Gall bladder	2 (1)
**ABO compatibility**		Fingernail	1 (0.5)
ABO match	123 (58.3)	Pancreas	1 (0.5)
Major mismatch	41 (19.4)	Bladder	1 (0.5)
Minor mismatch	38 (18)	Kidney	1 (0.5)
Bidirectional mismatch	9 (4.3)	Joint	1 (0.5)

Post-HSCT, post hematopoietic stem cell transplantation; AML, acute myelogenous leukemia; ALL, acute lymphoblastic leukemia; MDS, myelodysplastic syndrome; CML, chronic myelogenous leukemia; Haplo-HSCT, haploid hematopoietic stem cell transplantation; Sib-HSCT, sibling compatible hematopoietic stem cell transplantation; URD-HSCT, unrelated donor hematopoietic stem cell transplantation; BMT, bone marrow transplantation; PBSCT, peripheral blood stem cell transplantation; GVHD, graft-versus-host disease.

Age and post-HSCT time were expressed as mean ± standard deviation. Other data were presented as frequency (percentage).

The primary diseases included acute myelogenous leukemia (46%), acute lymphoblastic leukemia (29%), myelodysplastic syndrome (10%), chronic myelogenous leukemia (6%), and other types of blood diseases (9%), such as acute heterozygous cell leukemia, lymphoma, aplastic anemia, Fanconi anemia and granulocytic sarcoma.

50% cases underwent haploid hematopoietic stem cell transplantation (Haplo-HSCT), 39% cases underwent sibling compatible hematopoietic stem cell transplantation (Sib-HSCT), and 11% cases underwent unrelated donor hematopoietic stem cell transplantation (URD-HSCT). 49.6% cases were HLA-matched, while 50.4% cases were HLA-mismatched. 89% cases received graft from related donors, while 11% cases from unrelated donors. 58.3% had ABO-matched donors, while 19.4% had major mismatched donors, 18% had minor mismatched donors, 4.3% had bidirectional mismatched donors. 29% received male-to-male transplantation, 17% received female-to-female transplantation, 23% received male-to-female transplantation, and 31% received female-to-male transplantation. 7% used only bone marrow stem cells (BMT), 47% used only peripheral blood stem cells (PBSCT), while 46% used both, BMT and PBSCT.

A total of 208 patients reported with a medical history of systemic GVHD. Of these, 12 cases (6%) reported no other organ rejection was found. 196 cases (94%) had one or more organ involvement, including skin, oral mucosa, liver, gastrointestinal tract, lung, kidney, spleen, gall bladder, pancreas, bladder, joint and fingernail. The time of the occurrence of systemic rejection was provided in 172 patients, of whom 85 cases (49%) had a history of acute GVHD.

### Tear analysis

The median of TBUT was 0 second, and the median of tear secretion as detected by Schirmer I test was 2mm/5min.

### Corneal epitheliopathy

The median CFS score of 248 patients was 3 points. There were 70 cases (28%) in the C_0_ group, 56 cases (23%) in the C_1_ group, and 122 cases (49%) in the C_2_ group. Among 178 cases with positive corneal staining, the median CFS score was 4 points. There were 48 cases (27%) with surrounding punctate corneal staining, 45 (25%) with pupillary staining, 7 (4%) with staining fusing into clumps, 3 (2%) with filaments, 66 (37%) with pupillary staining along with staining fusing into clumps, and 9 (5%) with all these features. The main epitheliopathy of C_1_ group was punctate staining (86%), while that of C_2_ group was pupillary staining accompanied with staining fusing (53%).

As shown in **Table 3**, the grade of corneal epitheliopathy was associated with donor source (P<0.001), kinship (P=0.033), and HLA type (P<0.001). Patients in C_0_ group mainly underwent Haplo-HSCT (70%), the proportion was higher than that in C_1_ (39%) and C_2_ (43%). The proportion of Sib-HSCT was lower in C_0_ group (19%) than that in C_1_ (41%) and C_2_ (50%). The proportion of URD-HSCT was higher in C_1_ group (20%) than that in C_2_ (7%). There were statistical differences in donor source between C_1_ and C_2_, as the proportion of patients with related donor was higher in C_2_ group (93%) than that in C_1_ (80%). No statistical differences were found between C_0_ and C_1_, and C_0_ and C_2_. Compared with URD-HSCT, corneal epitheliopathy was more likely to be more severe in related donors (OR=3.287, 95%CI 1.315-8.215, P=0.011). Additionally, there were statistical differences in HLA matching analysis, as the proportion of HLA partially matching was higher in C_0_ (71%), while the proportion of HLA fully matching was higher in C_1_ (61%) and C_2_ (57%). No statistical differences were found between C_1_ and C_2_. Compared with HLA partially matching cases, corneal epitheliopathy was more likely to occur in HLA fully matching ones (OR=2.131, 95%CI 1.189-3.819, P=0.011) and may be more severe. The grade of corneal epitheliopathy was related to systemic GVHD (P=0.007), especially oral GVHD (P<0.001) and liver GVHD (P=0.002). The proportion of patients with systemic GVHD was higher in C_2_ (97%) than C_0_ (85%). No statistical differences were found between C_0_ and C_1_, and between C_1_ and C_2_. The proportion of patients with oral GVHD was lower in C_0_ (22%) than both, C_1_ (47%) and C_2_ (57%). No statistical differences were found between C_1_ and C_2_. The proportion of patients with liver GVHD was lower in C_0_ (28%) than both, C_1_ (53%) and C_2_ (57%). No statistical differences were found between C_1_ and C_2_. Corneal epitheliopathy was more likely to occur in patients with history of oral GVHD (OR=2.112, 95%CI 1.192-3.744, P=0.010) and liver GVHD (OR=1.849, 95%CI 1.049-3.258, P=0.034), and may be more severe. The grade of corneal epitheliopathy had no statistical relationship with gender (P=0.249), age (P=0.211), primary disease (P=0.933), ABO-compatibility (P=0.087), gender consistence (P=0.713), source of stem cell (P=0.822), acute GVHD (P=0.115), skin GVHD (P=0.960), gastrointestinal tract GVHD (P=0.381) and lung GVHD (p=0.179).

**Table 3:** Univariate analysis of ocular surface lesions.

Clinical Data	Corneal Epitheliopathy				Lid Margin Lesion				Meibomian Gland Loss			
	C_0_	C_1_	C_2_	P	L_0_	L_1_	L_2_	P	G_0_	G_1_	G_2_	P
**Gender**												
Male	37 (53)a	37 (66)a	77 (63)a	0.249	6 (37.5)a	55 (61)a	38 (70)a	0.058	25 (71)a	52 (58)a	28 (62)a	0.369
Female	33 (47)a	19 (34)a	45 (37)a		10 (62.5)a	35 (39)a	16 (30)a		10 (29)a	38 (42)a	17 (38)a	
**Age (y)**												
0-29	22 (31)a	26 (46)a	44 (36)a	0.211	8 (50)a	35 (39)a	15 (28)a	0.196	19 (54)a	27 (30)b	19 (42)a,b	**0.03 5**
≥30	48 (69)a	30 (54)a	78 (64)a		8 (50)a	55 (61)a	39 (72)a		16 (46)a	63 (70)b	26 (58)a,b	
**Primary disease**												
ALL	20 (29)a	15 (27)a	36 (29)a	0.933^c^	7 (44)a	27 (30)a	11 (20)a	0.412^c^	10 (29)a	28 (31)a	15 (33)a	0.779^c^
AML	32 (46)a	25 (45)a	56 (46)a		6 (37)a	42 (46.5)a	29 (54)a		13 (37)a	40 (44)a	21 (46)a	
CML	3 (4)a	3 (5)a	10 (8)a		0 (0)a	6 (6.5)a	3 (6)a		2 (6)a	9 (10)a	3 (7)a	
MDS	8 (11)a	6 (11)a	12 (10)a		0 (0)a	8 (9)a	7 (13)a		5 (14)a	8 (9)a	3 (7)a	
others	7 (10)a	7 (13)a	8 (7)a		3 (19)a	7 (8)a	4 (7)a		5 (14)a	5 (6)a	3 (7)a	
**Donor source**												
Haplo-HSCT	49 (70)a	22 (39)b	53 (43)b	**<0.001**	13 (81)a	40 (44)b	28 (52)a,b	**0.019^c^**	19 (54)a	38 (42)a	21 (47)a	0.687^c^
Sib-HSCT	13 (19)a	23 (41)b	61 (50)b		1 (6)a	42 (47)b	19 (35)a,b		14 (40)a	40 (45)a	18 (40)a	
URD-HSCT	8 (11)a,b	11 (20)b	8 (7)a		2 (13)a	8 (9)a	7 (13)a		2 (6)a	12 (13)a	6 (13)a	
**Kinship**												
Related	62 (89)a,b	45 (80)b	114 (93)a	**0.033**	14 (87.5)a	82 (91)a	47 (87)a	0.685^c^	33 (94)a	78 (87)a	39 (87)a	0.510^c^
Unrelated	8 (11)a,b	11 (20)b	8 (7)a		2 (12.5)a	8 (9)a	7 (13)a		2 (6)a	12 (13)a	6 (13)a	
**HLA**												
Fully match	20 (29)a	34 (61)b	69 (57)b	**<0.001**	2 (12.5)a	50 (56)b	26 (48)b	**0.006**	16 (46)a	52 (58)a	24 (53)a	0.474
Partially match	50 (71)a	22 (39)b	53 (43)b		14 (87.5)a	40 (44)b	28 (52)b		19 (54)a	38 (42)a	21 (47)a	
**ABO compatibility**												
ABO match	34 (51.5)a	22 (50)a	67 (66)a	0.087^c^	11 (69)a	43 (54)a	29 (63)a	0.937^c^	16 (52)a	45 (60)a	23 (61)a	0.390^c^
Major mismatch	16 (24)a	8 (18)a	17 (17)a		3 (19)a	18 (23)a	7 (15)a		10 (32)a	14 (19)a	6 (16)a	
Minor mismatch	15 (23)a	9 (21)a	14 (14)a		2 (12)a	14 (18)a	8 (18)a		2 (6)a	13 (17)a	7 (18)a	
Bidirectional mismatch	1 (1.5)a	5 (11)a	3 (3)a		0 (0)a	4 (5)a	2 (4)a		3 (10)a	3 (4)a	2 (5)a	
**Gender relationship**												
Male-to-male	21 (30.4)a	14 (27)a	33 (28)a	0.713	4 (25)a	26 (30)a	16 (31)a	0.394^c^	13 (37)a	20 (24)a	12 (29)a	0.652
Female-to-female	20 (29)a	10 (20)a	25 (22)a		5 (31)a	19 (22)a	9 (17)a		7 (20)a	19 (23)a	11 (26)a	
Male-to-female	16 (23.2)a	18 (35)a	40 (34)a		2 (13)a	27 (32)a	20 (38.5)a		12 (34)a	28 (33)a	13 (31)a	
Female-to-male	12 (17.4)a	9 (18)a	18 (16)a		5 (31)a	14 (16)a	7 (13.5)a		3 (9)a	17 (20)a	6 (14)a	
**Source of stem cell**												
BMT	5 (8)a	4 (8)a	8 (7)a	0.822^c^	0 (0)a	5 (6)a	3 (6)a	0.158^c^	2 (6)a	5 (6)a	5 (12)a	0.325^c^
PBSCT	34 (52)a	23 (45)a	50 (44)a		12 (75)a	40 (46)a,b	19 (38)b		12 (35)a	43 (52)a	20 (48)a	
BMT+PBSCT	26 (40)a	24 (47)a	56 (49)a		4 (25)a	41 (48)a	28 (56)a		20 (59)a	34 (42)a	17 (40)a	
**Systemic GVHD**												
With	46 (85)a	46 (98)a,b	104 (97)b	**0.007^c^**	9 (82)a	74 (90)a,b	48 (100)b	**0.013^c^**	29 (91)a	68 (92)a	40 (100)a	0.126^c^
Without	8 (15)a	1 (2)a,b	3 (3)b		2 (18)a	8 (10)a,b	0 (0)b		3 (9)a	6 (8)a	0 (0)a	
**Acute GVHD**												
With	15 (36)a	22 (56)a	48 (53)a	0.115	3 (37.5)a	34 (48)a	19 (46)a	0.877^c^	15 (50)a	32 (51)a	16 (44)a	0.823
Without	27 (64)a	17 (44)a	43 (47)a		5 (62.5)a	37 (52)a	22 (54)a		15 (50)a	31 (49)a	20 (56)a	
**Organs with GVHD**												
Skin	40 (74)a	34 (72)a	77 (72)a	0.960	9 (82)a, b	54 (66)b	42 (87.5)a	**0.019^c^**	21 (66)a	51 (69)a	33 (82.5)a	0.204
Oral mucosa	12 (22)a	22 (47)b	61 (57)b	**<0.001**	1 (9)a	43 (52)b	26 (54)b	**0.019**	12 (37.5)a	39 (53)a	19 (47.5)a	0.355
Liver	15 (28)a	25 (53)b	61 (57)b	**0.002**	2 (18)a	42 (51)a	27 (56)a	0.073	13 (41)a	43 (58)a	19 (47.5)a	0.216
Gastrointestinal tract	9 (17)a	12 (26)a	28 (26)a	0.381	1 (9)a	16 (20)a	14 (29)a	0.282^c^	8 (25)a, b	7 (9)b	13 (32.5)a	**0.007**
Lung	5 (9)a	10 (21)a	13 (12)a	0.179	1 (9)a	10 (12)a	7 (15)a	0.923^c^	5 (16)a	13 (18)a	4 (10)a	0.595^c^

AML, acute myelogenous leukemia; ALL, acute lymphoblastic leukemia; MDS, myelodysplastic syndrome; CML, chronic myelogenous leukemia; Haplo-HSCT, haploid hematopoietic stem cell transplantation; Sib-HSCT, sibling compatible hematopoietic stem cell transplantation; URD-HSCT, unrelated matched donor hematopoietic stem cell transplantation; BMT, bone marrow transplantation; PBSCT, peripheral blood stem cell transplantation; GVHD, graft-versus-host disease.

Comparisons among groups (C_0_, C_1_, C_2_; L_0_, L_1_, L_2_; G_0_, G_1_, G_2_; respectively) were using the Chi-square test unless indicated.

The letter labeling method (a, b) was used. If there is a common letter between two groups, it indicates that there is no significant difference. If there is no common letter, it indicates a significant difference.

Data were presented as frequency (percentage).

c. Fisher exact test.

The bold value: A P-value of <0.05 was considered to be statistically significant.

### Lid margin lesions

Assessment of lid margin lesions was done for 160 of 248 patients, as 160 cases had complete data. The median total lid margin lesion score was 6 points. There were 16 cases (10%) in the L_0_ group, 90 cases (56%) in the L_1_ group, and 54 cases (34%) in the L_2_ group.

As shown in **Table 3**, the grade of lid margin lesions was associated with donor source (P=0.019) and HLA type (P=0.006). Patients in L_0_ group mainly underwent Haplo-HSCT (81%), while in L_1_ group mainly underwent Sib-HSCT (47%). The proportion of patients who underwent HLA partially matching transplantation was higher in L_0_ group (87.5%) than that in L_1_ (44%) and L_2_ (52%). No statistical difference was found between L_1_ and L_2_. Taken L_0_ group as a reference, patients underwent HLA fully matching transplantation were more likely to develop mild lid margin lesions than partially matching ones (OR=5.478, 95%CI 1.074-27.932, P=0.041). Moreover, the grade of lid margin lesions was associated with systemic GVHD (P=0.013), especially skin GVHD (P=0.019) and oral GVHD (P=0.019). The proportion of patients with systemic GVHD was higher in L_2_ (100%) than L_0_ (82%). No statistical difference was found between L_0_ and L_1_, and between L_1_ and L_2_. The proportion of patients with skin GVHD was lower in L_1_ (66%) than L_2_ (87.5%). No statistical difference was found between L_0_ and L_1_, and between L_0_ and L_2_. The proportion of patients with oral GVHD was lower in L_0_ (9%) than both, L_1_ (52%) and L_2_ (54%). No statistical difference was found between L_1_ and L_2_. Taken L_0_ group as a reference, patients with oral GVHD were more likely to develop mild lid margin lesions (OR=11.488, 95%CI 1.365-96.666, P=0.025) and severe lid margin lesions (OR=11.149, 95%CI 1.302-95.466, P=0.028).The grade of lid margin lesions had no statistical relationship with gender (P=0.058), age (P=0.196), primary disease (P=0.412), kinship (P=0.685), ABO-compatibility (P=0.937), gender consistence (P=0.394), source of stem cell (P=0.158), acute GVHD (P=0.877), liver GVHD (P=0.073), gastrointestinal tract GVHD (P=0.282) and lung GVHD (p=0.923).

### Meibomian gland loss

Assessment of meibomian gland loss was done for 170 of 248 patients, as 170 cases had complete data. The median meibomian gland loss score was 2 points. There were 35 cases (21%) in the G_0_ group, 90 (53%) in the G_1_ group, and 45 (26%) in the G_2_ group.

As shown in **Table 3**, the grade of meibomian gland loss was associated with age (P=0.035) and gastrointestinal tract GVHD (P=0.007). The proportion of patients younger than 30 years old was higher in G_0_ group (54%) than G_1_ (30%). No statistical difference was found between G_0_ and G_2_, and between G_1_ and G_2_. The proportion of patients with gastrointestinal tract GVHD was lower in G_1_ group (9%) than G_2_ (32.5%). No statistical difference was found between G_0_ and G_1_, and between G_0_ and G_2_. Taken G_2_ group as a reference, the history of gastrointestinal rejection was less likely to be found in the mild lesion group than in the severe lesion group (OR=0.224, 95%CI 0.078-0.646, P=0.006). There was no statistical relationship between the grade of meibomian gland loss and gender (P=0.369), primary disease (P=0.779), donor source (P=0.687), kinship (P=0.510), HLA type (P=0.474), ABO-compatibility (P=0.390), gender consistence (P=0.652), source of stem cell (P=0.325), acute GVHD (P=0.823), skin GVHD (P=0.204), oral GVHD (P=0.355), liver GVHD (P=0.216), and lung GVHD (p=0.595).

### Correlations among corneal epitheliopathy, lid margin lesions and meibomian gland loss

As shown in **Table 4**, the grade of corneal epitheliopathy was associated with the scores of lid margin hyperemia (P<0.001), meibum quality (P=0.006), meibum secretion (P<0.001), gland obstruction (P<0.001), and total lid margin score (P<0.001). The total lid margin lesion score was lower for C_0_ group than for both, C_1_ (P=0.006) and C_2_ (P<0.001) groups. No statistical difference was found between C_1_ and C_2_. The hyperemia score was higher for C_2_ group than for both, C_0_ (P<0.001) and C_1_ (P=0.004) groups. No statistical difference was found between C_0_ and C_1_. The meibum quality score was lower for C_0_ group than for both, C_1_ (P=0.016) and C_2_ (p=0.020) groups. No statistical difference was found between C_1_ and C_2_. The meibum secretion score was lower for C_0_ group than for both, C_1_ (P=0.002) and C_2_ (P<0.001) groups. No statistical difference was found between C_1_ and C_2_. The gland obstruction score was lower for C_0_ group than for both, C_1_ (P=0.010) and C_2_ (P<0.001) groups. No statistical difference was found between C_1_ and C_2_.

**Table 4:** Correlation among ocular surface lesions.

Parameters	Corneal Epitheliopathy				Lid Margin Lesion				Meibomian Gland Loss			
	C_0_	C_1_	C_2_	P	L_0_	L_1_	L_2_	P	G_0_	G_1_	G_2_	P
**Corneal**												
CFS	–	–	–	–	0 (0,0)a	4 (0,5)b	4 (3,5)b	**<0.001**	3 (0,4)a	4 (1.75,5)a	4 (3,5)a	0.189
**Lid Margin**												
Hyperemia	0 (0,1)a	1 (0,2)a	2 (1,3)b	**<0.001**	–	–	–	–	1 (0,2)a	1 (0,2)a	1 (0,3)a	0.491
Irregularity	0 (0,1)a	0 (0,0)a	0 (0,1)a	0.353	–	–	–	–	0 (0,0)a	0 (0,1)a,b	0 (0,2)b	**0.003**
Meibum quality	0 (0,1)a	1 (0,1)b	1 (0,2)b	**0.006**	–	–	–	–	0 (0,1)a	0.5 (0,1)a	1 (0,2)a	0.161
Meibum secretion	0 (0,1)a	2 (1,2)b	2 (1,3)b	**<0.001**	–	–	–	–	1 (1,2)a	2 (1,2)a	2 (1,2)a	0.623
Gland obstruction	0 (0,1)a	2 (1,2.5)b	2 (1,3)b	**<0.001**	–	–	–	–	2 (0.25,3)a	2 (1,3)a	2 (1,3)a	1.000
Total score	2.5 (0,5.25)a	6 (4.5,8)b	7 (5,10)b	**<0.001**	–	–	–	–	5 (3,7.75)a	6 (4,8)a	6.5 (4,9.75)a	0.134
**Meibomian Gland**												
Gland loss	2 (0,3)a	2 (1,3.5)a	2 (1,4)a	0.647	2 (0.25,2.75)a	2 (1,4)a	3 (2,5)a	0.113	–	–	–	–

Comparisons among groups (C_0_, C_1_, C_2_; L_0_, L_1_, L_2_; G_0_, G_1_, G_2_; respectively) were using the Kruskal-Wallis test and pair-wise comparations.

The letter labeling method (a, b) was used. If there is a common letter between two groups, it indicates that there is no significant difference; If there is no common letter, it indicates a significant difference.

Data were presented as median (inter-quartile range).

The bold value: A P-value of <0.05 was considered to be statistically significant.

The grade of meibomian gland loss was associated with the score of lid margin irregularity (P=0.003). The score of lid margin irregularity was lower for G_0_ group than for G_2_ (P=0.002) groups. No statistical difference was found between C_0_ and C_1_, and between C_1_ and C_2_.

## Discussion

Allogeneic HSCT is a common treatment for blood diseases. However, GVHD is a frequent complication associated with it, and oGVHD is seen in 60% to 90% of patients with GVHD. Ocular GVHD most frequently affects the ocular surfaces, and results in foreign body sensation, reduces visual quality and affects daily life (8). Currently, the diagnosis of oGVHD is mainly based on tear secretion level, as detected by Schirmer test, and is assisted by corneal epitheliopathy. However, almost all patients who visit the ophthalmology department after HSCT have dry eye symptoms with relatively low tear secretion level. Moreover, the Schirmer test is a non-specific test for oGVHD. Hence, it is difficult to evaluate the severity of dry eye associated with oGVHD using the Schirmer test and TBUT (16, 17). Therefore, the diagnosis and grading of oGVHD-associated dry eye requires comprehensive evaluation of several ocular surface lesions.

In this study, 72% of the patients were identified having varying degrees of corneal epitheliopathy after HSCT, and the median fluorescence staining score was 4 points. Most of them had pupillary staining and/or staining fusing into clumps, which indicates severe corneal epitheliopathy. Patients with oGVHD dry eye usually have more severe corneal epitheliopathy. In severe cases, corneal stromatolysis and corneal perforation may occur (6). Except KCS, which leads to corneal epitheliopathy, lesions of lid margin and meibomian gland can also occur in patients with oGVHD dry eye, manifesting as blepharitis, MGD and other ocular surface diseases. MGD can serve as the most important symptom of eyelid dysfunction, and an early manifestation of oGVHD (17). Eyelid changes are the earliest indication of involvement of eyes in diseases for some animal models of GVHD (18). In this study, we conducted a detailed evaluation of the lid margin in patients after HSCT. We identified that most of the patients had different degrees of lid margin hyperemia, meibomian gland secretion disorder, and plugging of gland orifices. According to infrared imaging results of meibomian gland, 79% of the patients had different degrees of meibomian gland loss after HSCT. Of these, majority had an area loss of 1/3 to 2/3 of the total meibomian gland area. Moreover, the lid margin was more irregular in patients with severe meibomian gland loss. However, the degree of meibomian gland loss is also correlated with age, and may be more severe in older patients (15). In addition, we identified a positive correlation between the lid margin lesion score and corneal epitheliopathy score, indicating that corneal epitheliopathy was relatively more severe in patients with severe lid margin lesions. Since patients with GVHD are often followed up by hematologists, early detection of oGVHD keratopathy is difficult. As observation of lid margin lesions is more convenient and non-invasive, paying more attention to lid margin can contribute to timely diagnosis of severe oGVHD dry eye, and to predict disease progression.

The efficacy of HSCT mainly depends on T-cell-driven graft-versus-tumor reaction by donor T-cells that attack various tissues and organs of the recipient, such as skin, liver and gastrointestinal tract, leading to GVHD (19, 20). Thus, donor selection influences the occurrence and severity of oGVHD after HSCT. This study analyzes the donor-recipient information, such as donor type (Haplo-HSCT, Sib-HSCT or URD-HSCT), kinship (related donor or unrelated donor from Chinese bone marrow bank), HLA (fully or partially match), ABO blood type (ABO-match, major mismatch, minor mismatch or bidirectional mismatch), and gender (male-to-male, female-to-female, male-to-female or female-to-male), in order to assess the correlation between donor source and ocular surface lesions after HSCT.

In this study, we found that the severity of corneal epitheliopathy in oGVHD was associated with donor source, kinship and HLA compatibility. The severity of lid margin lesions was associated with donor source and HLA compatibility. In contrast, the severity of meibomian gland loss was not significantly associated with these parameters. At present, the main criteria for donor selection for patients with hematologic diseases is HLA compatibility. HLA-matched sibling donors (Sib-HSCT) are the first choice (21), followed by HLA-matched unrelated donors. Whereas, only about 30% of patients have fully matched sibling donors in Europe. This proportion is even more erratic in America. Moreover, the probability of Sib-HSCT decreases as fertility declines, since it becomes difficult to find a HLA-matched sibling donor. Additionally, there are certain restrictions on the selection of unrelated donors due to race and laws of different nations (22). Whether HLA matched or mismatched, acute or chronic GVHD can occur after HSCT (23, 24). In the present study, all subjects belonged to Chinese Han population. 50% of the patients underwent Haplo-HSCT, followed by Sib-HSCT (39%). The proportion of HLA partially matching was high in the no corneal epitheliopathy group (71%) and no lid margin lesion group (87.5%). In contrast, the proportion of HLA fully matching was higher in the mild and severe groups. Haplo-HSCT was more common in the no corneal epitheliopathy group (70%) and no lid margin lesion group (81%), obviously higher than groups with lesions. These results indicate that the occurrence of corneal epitheliopathy and lid margin lesions may relatively lesser after HLA partially matching transplantation, and the lesions may be milder. Previously, since the bidirectional allorecognition immune response between donor-recipient HLA disparities, Haplo-HSCT was considered to result in severe GVHD and a high transplant-related mortality (25). Nowadays, owing to the development of novel graft manipulation and prophylaxis of GVHD, the results of Haplo-HSCT have improved significantly (26). According to the reports of the European Society for Blood and Marrow Transplantation, the use of haplo-identical donors for various blood diseases has increased rapidly in recent years (27). In addition, across all transplantation types, the greatest reduction in non-relapse mortality and decrease in GVHD incidence have been observed among the recipients of Haplo-HSCT, which may relate to the graft cell processing, control of bidirectional T cell alloreactivity, GVHD prophylaxis and use of cyclophosphamide after transplantation (21, 22). However, different studies utilized different methods to prevent GVHD before and after Haplo-HSCT, making it complicated to compare and analyze the occurrence of GVHD after HSCT.

When studying the correlation between donor-recipient relationship and degree of corneal epitheliopathy, we found that there were more related donors in severe lesion group (93%) than mild lesion group (80%). This indicates that the severity of corneal epitheliopathy in oGVHD may be more severe in case of related donors. Few studies have reported that incidence of oGVHD is higher in related donors than in unrelated donors, which may be attributed to the use of anti-T-cell globulin (ATG) in unrelated donor grafts for precondition (23). Due to the reaction of T-cells, patients receiving URD-HSCT are more likely to develop GVHD than those receiving Sib-HSCT with the same precondition. Hence, pretreatment is usually performed to reduce T-cells in the graft before transplantation in URD-HSCT. Depletion of T-cells in the graft may increase the risk of infection and relapse after HSCT. While patients with *in vivo* T-cell depletion using ATG show reduced incidence of acute or chronic GVHD, without increasing mortality and affecting overall survival (28). Many studies conducted in Europe and Australia have proved that compared with HLA-matched sibling transplantation, the occurrence of chronic GVHD was lower in URD-HSCT with ATG (29, 30). Thus, the correlation between the selection of donor and the occurrence and severity of oGVHD needs to be further studied on the premise of controlling precondition before transplantation.

Many studies have reported a correlation between systemic GVHD and ocular GVHD. Ocular GVHD is more likely to occur in patients with oral, skin and liver GVHD, the reason being that mucous membranes on the surface of lacrimal glands, meibomian glands, salivary glands and hepatic ducts are all targets of T-cells and other inflammatory cells (1, 31). Our study found that corneal epitheliopathy, lid margin lesions and meibomian gland loss after HSCT were all related to systemic GVHD. Patients with systemic GVHD, especially ones with oral or liver GVHD are likely to develop corneal epitheliopathy. Patients with oral rejection history were more likely to develop lid margin lesions. Patients with history of skin GVHD may develop more severe lid margin lesions than those without history of GVHD. The loss of meibomian gland is associated with gastrointestinal tract GVHD. The proportion of gastrointestinal tract GVHD history was lower in patients with mild gland loss than those with severe loss. No correlation was found between the history of acute GVHD and the development of ocular GVHD in this study. Notably, ocular GVHD develops prior to lesions of other tissues and organs, and can even occur singly. In our study, about 6% of the patients reported ocular symptoms without rejection of other organs after HSCT.

The limitation of this study is that, as a retrospective study, there may be some bias in the collection of cases. Meanwhile, the collection process of cases was long and difficult, and the completeness of medical record was still lacking. In addition, other factors which may influence the analysis results, such as differences in treatment plans, prophylaxis pre and post HSCT towards different types of blood disease, haven’t been statistically analyzed in this study. In our following study, we will collect the medical history of the patients as completely as possible, and evaluate the ocular condition pre and post transplantation, in order to find out more features of the ocular surface after HSCT and the risk factors causing these lesions.

## Conclusion

In summary, the occurrence and development of ocular GVHD are mostly accompanied by the history of systemic GVHD. While in few cases, ocular surface lesions related to GVHD can be observed prior to the rejection of other tissues and organs. After allogeneic HSCT, corneal epithelium and lid margin can get damaged, and the morphology and function of meibomian gland can change to different degrees. Severe corneal epitheliopathy occurs in patients with severe lid margin lesions in ocular GVHD. Focusing attention to changes in lid margin can contribute to timely detection of severe corneal epitheliopathy with oGVHD. Patients who underwent HLA partially matching transplantation exhibited milder corneal epitheliopathy and lid margin lesions.

## Data availability statement

The original contributions presented in the study are included in the article/supplementary material. Further inquiries can be directed to the corresponding author.

## Ethics statement

The studies involving human participants were reviewed and approved by the First Affiliated Hospital of Soochow University (No.2022-235). Written informed consent to participate in this study was provided by the participants’ legal guardian/next of kin.

## Author contributions

X-FZ defined research topics and discussed analysis. X-YZ and Z-TS analyzed data, illustrated the results and wrote the manuscript. YX, Y-RR, and Y-JC contributed to the data collection and reference collection. FC, XM, and X-WT contributed to the data collection and statistical analysis.

## Conflict of interest

The authors declare that the research was conducted in the absence of any commercial or financial relationships that could be construed as a potential conflict of interest.

## Publisher’s note

All claims expressed in this article are solely those of the authors and do not necessarily represent those of their affiliated organizations, or those of the publisher, the editors and the reviewers. Any product that may be evaluated in this article, or claim that may be made by its manufacturer, is not guaranteed or endorsed by the publisher.

## Abbreviations

GVHD, graft-versus-host disease; oGVHD, ocular graft-versus-host disease; HSCT, hematopoietic stem cell transplantation; NIH, National Institutes of Health; HLA, human leukocyte antigen; KCS, Keratoconjunctivitis sicca; MGD, Meibomian gland dysfunction; TBUT, tear break-up time; CFS, corneal fluorescence staining; Haplo-HSCT, haploid hematopoietic stem cell transplantation; Sib-HSCT, sibling compatible hematopoietic stem cell transplantation; URD-HSCT, unrelated donor hematopoietic stem cell transplantation; Post-HSCT, post hematopoietic stem cell transplantation; BMT, bone marrow stem cells; PBSCT, peripheral blood stem cells; ATG, anti-T-cell globulin; AML, acute myelogenous leukemia; ALL, acute lymphoblastic leukemia; MDS, myelodysplastic syndrome; CML, chronic myelogenous leukemia.
